# Phylogenetic Analyses Reveal Monophyletic Origin of the Ergot Alkaloid Gene *dmaW* in Fungi

**DOI:** 10.4137/ebo.s2633

**Published:** 2009-06-04

**Authors:** Miao Liu, Daniel G. Panaccione, Christopher L. Schardl

**Affiliations:** 1 201 F Plant Science Bldg. University of Kentucky, Lexington, KY 40546; 2 Division of Plant and Soil Sciences, West Virginia University, PO Box 6108, Morgantown, WV 26506-6108; 3 Biodiversity (Mycology and Botany), Eastern Cereal and Oilseed Research Centre, Agriculture and Agri-Food Canada, 960 Carling Avenue, Ottawa, Ontario K1A 0C6. Email:schardl@email.uky.edu

**Keywords:** mycotoxins, 4-γ,γ-dimethylallyltryptophan synthase gene, Clavicipitaceae, Trichocomaceae, amino acid sequence, GBlocks, gene phylogeny

## Abstract

Ergot alkaloids are indole-derived mycotoxins that are important in agriculture and medicine. Ergot alkaloids are produced by a few representatives of two distantly related fungal lineages, the Clavicipitaceae and the Trichocomaceae. Comparison of the ergot alkaloid gene clusters from these two lineages revealed differences in the relative positions and orientations of several genes. The question arose: is ergot alkaloid biosynthetic capability from a common origin? We used a molecular phylogenetic approach to gain insights into the evolution of ergot alkaloid biosynthesis. The 4-γ,γ-dimethylallyltryptophan synthase gene, *dmaW*, encodes the first step in the pathway. Amino acid sequences deduced from *dmaW* and homologs were submitted to phylogenetic analysis, and the results indicated that *dmaW* of *Aspergillus fumigatus* (mitosporic Trichocomaceae) has the same origin as corresponding genes from clavicipitaceous fungi. Relationships of authentic *dmaW* genes suggest that they originated from multiple gene duplications with subsequent losses of original or duplicate versions in some lineages.

## Introduction

Ergot alkaloids (EA) are a group of mycotoxins causing toxicoses in humans and animals.^1^–^3^ Producers of EA include plant pathogens in the genus *Claviceps*, and some grass endophytes in the genera *Epichloë*, *Neotyphodium* and *Balansia*.^2^ These genera belong to the family Clavicipitaceae (order Hypocreales, phylum Ascomycota). EA are also produced by the common airborne fungus *Aspergillus fumigatus*, a species distantly related with clavicipitaceous fungi, belonging to the family Trichocomaceae (order Eurotiales, phylum Ascomycota).^4^ In the same family, some *Penicillium* species also produce EA.^5^,^6^ Relatively few species within these families produce ergot alkaloids and only one representative of the lineages in between the two orders containing these families has been reported to produce ergot alkaloids.^7^,^8^ Because of their significant impacts on human health and agriculture, the biochemistry and biosynthesis pathway of ergot alkaloids have attracted much attention and effort.^3^ Certain genes involved in the biosynthesis pathway have been characterized.^9^–^15^ Clustered arrangements of EA biosynthesis genes have been observed in *A. fumigatus*,^10^ *Claviceps fusiformis*,^8^ and *Claviceps purpurea*.^14^ A gene, *dmaW*, common to all these clusters, encodes 4-γ,γ-dimethylallyltryptophan (DMATrp) synthase, which has been demonstrated to be the first pathway specific step and has the key regulatory function for EA biosyntheses in *Claviceps* spp.^15^–^17^ Following the cloning of *dmaW* from *C. fusiformis* SD58,^18^ a gene cluster likely encoding enzymes of EA biosynthesis was identified by sequence analysis in the ergot fungus, *C. purpurea* P1.^14^ This cluster was thereafter designated *EAS* (ergot alkaloid synthesis).^2^ Within the *EAS* cluster, four genes with known functions were named according to their functions, and the other seven genes with unknown functions were named *easA* and *easC* to *easH*. Homologs of nine *EAS*-cluster genes have been found in *C. fusiformis* SD58,^18^,^19^ and eight homologs have been found clustered in the *A. fumigatus* genome^10^ (Supplementary Fig. 1).

Based on the sequence similarity of *dmaW* genes and general clustering of other hypothetical *EAS* genes, Coyle and Panaccione^10^ proposed that EA biosynthesis in *A. fumigatus* has a common origin with that in clavicipitaceous fungi. However, arrangement of the cluster genes in *A. fumigatus* is drastically different from the *EAS* clusters in *C. purpurea* and *C. fusiformis*.^2^,^8^ This difference in gene arrangements, and the absence of ergot alkaloids in the lineages between the two groups of EA-producing fungi, raise the question of whether the *dmaW* genes in clavicipitaceous and trichocomaceous fungi are true orthologs, homologs due to speciation. If the genes are truly orthologous, then multiple gene recombinations and inversions must have happened in either (or both) of the lineages after the divergence from their common origin, and the lineages between them have lost these genes. The alternative would be that the *A. fumigatus EAS* genes might have evolved independently from different gene duplication events.

Recently, multiple genes similar or related to *dmaW* have been characterized by cloning and over-expression approaches. Examples include genes for brevianamide F prenyl transferase (FtmPT1) that converts brevianamide to tryprostatin in *A. fumigatus*,^20^ reverse prenyl transferase FGAPT1 catalyzing the final step in the biosynthesis of fumigaclavine C in the same fungus,^21^ TdiB with indole alkaloid biosynthetic ability in *A. nidulans*,^22^ as well as *SirD*, involved in the biosynthesis of an epipolythiodioxopiperazine (ETP) from *Leptosphaeria maculans*.^23^ The available sequences of these genes allow us to look into the evolutionary relationships of *dmaW* and related genes by a phylogenetic approach.

In this study, we conduct molecular phylogenetic analysis of inferred protein sequences to test whether the known DMATrp synthases in fungi have a single origin or multiple origins. A monophyletic pattern is expected for the single origin hypotheses; whereas a polyphyletic pattern is expected for multiple origins.

## Materials and Methods

Protein sequences derived from *dmaW* genes of clavicipitaceous fungi and *A. fumigatus* were obtained from previous studies.^10^,^15^,^18^ Protein sequences of FtmPT1, FGAPT1, and the *sirD* product were downloaded from GenBank (Table 1). The protein sequences from *dmaW* homologs were obtained by BLAST of the nonredundant protein sequence database in GenBank with the protein product deduced from *dmaW* from *Neotyphodium lolii* and *A. fumigatus*; sequences with relatively high similarity scores and low E-value (<3e^−17^) were selected (Table 1).

### Protein sequence matrix

Due to the high divergence of amino acid sequences, we used the program MAFFT ver.5.8^24^ to align them. The alignment was conducted through the web server (http://align.genome.jp/mafft/). The FFT-NS-i and E-INS-i alignment strategies, iterative refinement method, were used to enhance the accuracy of the alignment. The scoring matrix (for amino acid sequences) was selected from six options by comparing fitnesses of the trees in the preliminary analysis. With the scoring matrix selected, we set gap opening penalty (OP = 1.0, 2.0, 3.0) and gap extension penalty (offset value as shown in the program, OF = 0.0, 0.5, 1.0), and again compared the overall fitness of the resulting trees in more preliminary analyses. Parameters resulting in trees of high fitness were set in the final alignment. The alignments by MAFFT were submitted to the program Gblocks 0.91b,^25^ to eliminate the highly diverged regions and retain the conserved regions for phylogenetic analysis. Low stringency options were selected to obtain blocks.

### Phylogenetic analysis

The protein matrices of ten operational taxonomic units (OTUs, six authentic *dmaW* and four related genes of known functions) resulting from MAFFT and Gblocks screening and comprised of the conserved regions of the protein alignment, were submitted separately to phylogenetic analysis in PAUP* 4.0b10.^26^ Parsimony analyses were conducted using exhaustive search. All characters had equal weight and gaps were treated as missing data.

In order to exclude the possible bias caused by insufficient OTUs, we included multiple potential homologs of *dmaW* products, which were obtained by BLAST search, in a more extensive phylogenetic analysis. Both conserved protein regions and the whole protein sequence were used in the separate analyses. Parsimony analysis was conducted with a heuristic search with TBR (tree bisection and reconnection) branch-swapping and 100 replicates of random sequence addition. Bootstrapping analysis was based on 1000 replicates of a full heuristic search, each with 20 replicates of a random addition sequence, and tree bisection reconnection (TBR) swapping was selected and re-arrange limit was set to 5000 per replicate.

Bayesian inferences (BI) were performed using MrBayes 3.0B5^27^ to analyze individual data sets. The prior for amino acid model was set as mixed to allow model jumping between fixed-rate aminoacid models. Maximum likelihood analysis (ML) was performed using PHYML online server^28^ with the following settings: substitution model as JTT, transition/transversion ratio and gamma distribution as estimated by the program, bootstrap datasets 500.

## Results

### Protein sequence matrix

To choose the appropriate scoring matrix in the amino acid sequence alignment, we compared the overall fitness of the trees based on the alignments of six different scoring matrices in two strategies (Table 2). Setting scoring matrix as JTT200 resulted in the highest consistency index (CI), lowest homoplasy index (HI), relatively high retention index (RI), and shorter trees, thus we choose JTT200 for alignments. In the combination of various OP and OF in the alignment, OP/OF = 1.0/0.0 resulted in three shortest parsimonious trees with a relatively high CI, RI and low HI (Table 3).

For the protein sequences derived from six *dmaW* and four related genes of known functions, an analysis in which the scoring matrix was set as JTT200, OP as 1.0, and OF as 0.0 resulted in an alignment with 653 characters. The resulting alignment was put into Gblocks 0.91b to screen for conserved regions. The number of characters (amino acid positions) retained was 308. For the matrix from the extended OTU set (34 protein sequences), 933 characters resulted from MAFFT alignment, and 155 characters were retained after GBlocks screening.

### Phylogenetic relationships

**Figure 1:**
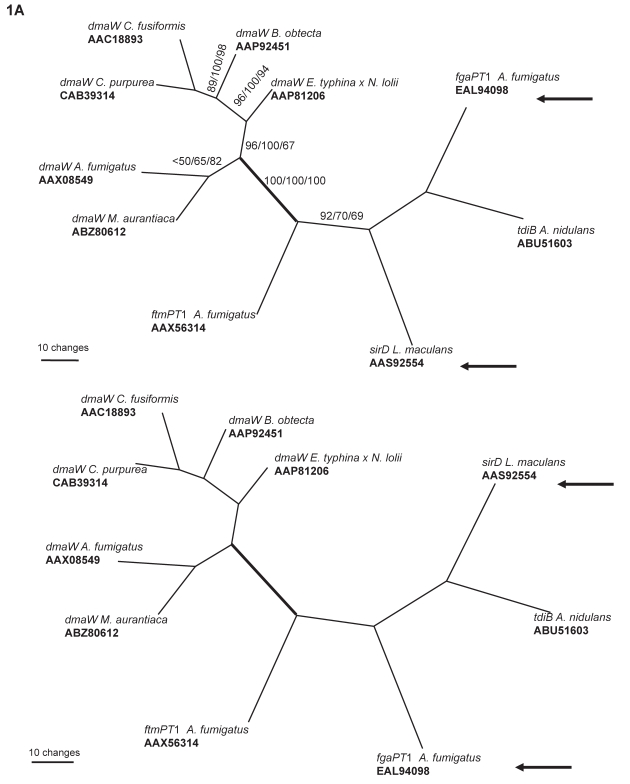

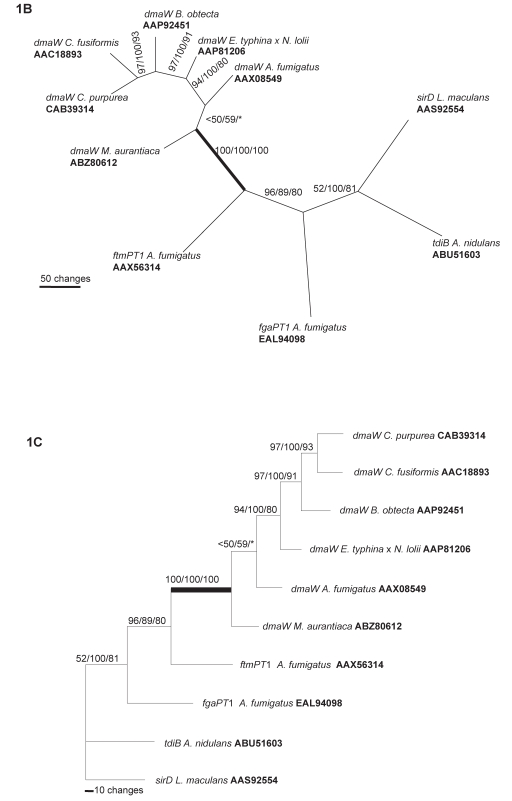
The most parsimonious trees relating amino acid sequences of the deduced products of six *dmaW* genes and four related genes with known functions. **A**) Tree based on whole gene region. Of 653 aligned characters, 250 characters are informative. Length = 1521, CI = 0.870, RI = 0.503. Arrows show the discrepancies of the two most parsimonious trees. **B** and **C**) Trees based on relatively conserved gene regions screened by GBlocks. Of 308 total aligned characters, 166 characters are informative. Length = 934, CI = 0.864, RI = 0.512. Products of *dmaW* genes formed a monophyletic group with 100% bootstrap support. B. Unrooted tree; C. Phylogram rooted by choosing the non-*dmaW* gene products as the outgroup. numbers on branches indicate bootstrap percentage of MP/posterior probabilities of BI/bootstrap percentage of ML; *indicates that particular branch does not exist in the analysis. The thick branch separates of the outgroup from the ingroup.

For the data set comprised of ten OTUs, parsimony analyses for the whole regions resulted in two most parsimonious trees (Fig. 1A). The two tree topologies differed in the order of branches ranches to *sirD L. maculans* and *fgaPT1 A. fumigatus*. The analysis of the conserved regions resulted in one most parsimonious tree. The tree topology differed from those of whole gene regions in the order of divergence of *dmaW* of *A. fumigatus* and *Malbranchea aurantiaca* (Fig. 1B). The known, authentic sequences of DMATrp synthases formed a monophyletic clade with strong bootstrap support. Defining as an outgroup those prenyl transferases known or likely to catalyze production of other products (4 OTUs), the most basal divergence separated the *dmaW* gene of *M. aurantiaca* and *A. fumigatus* from those of the Clavicipitaceae (Fig. 1C).

**Figure 2:**
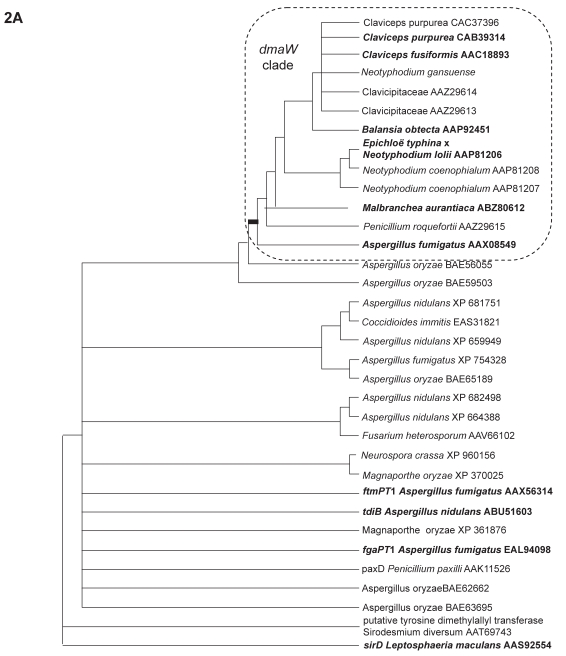

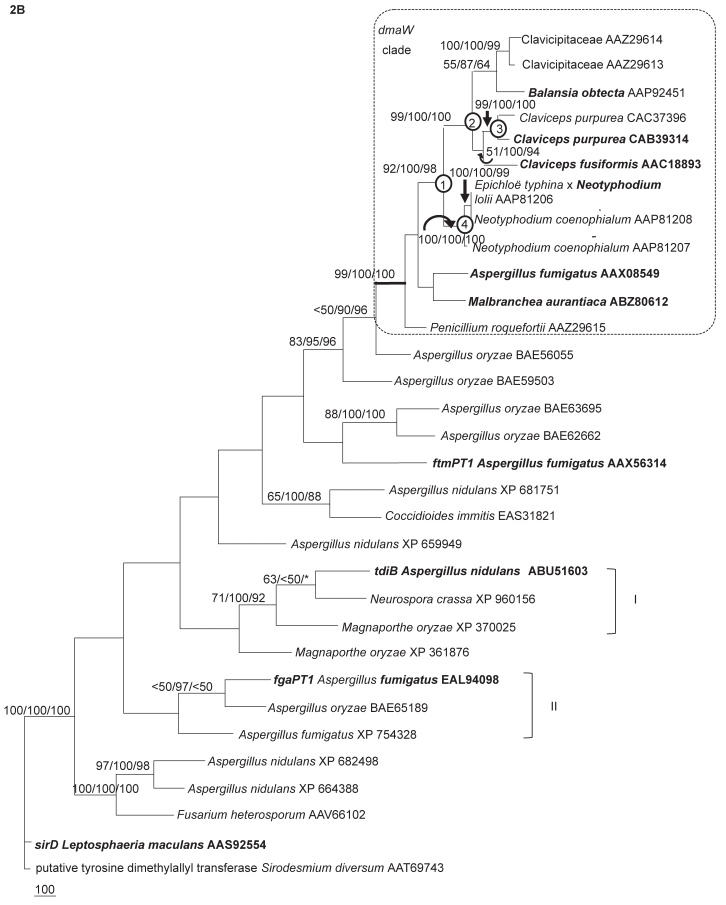

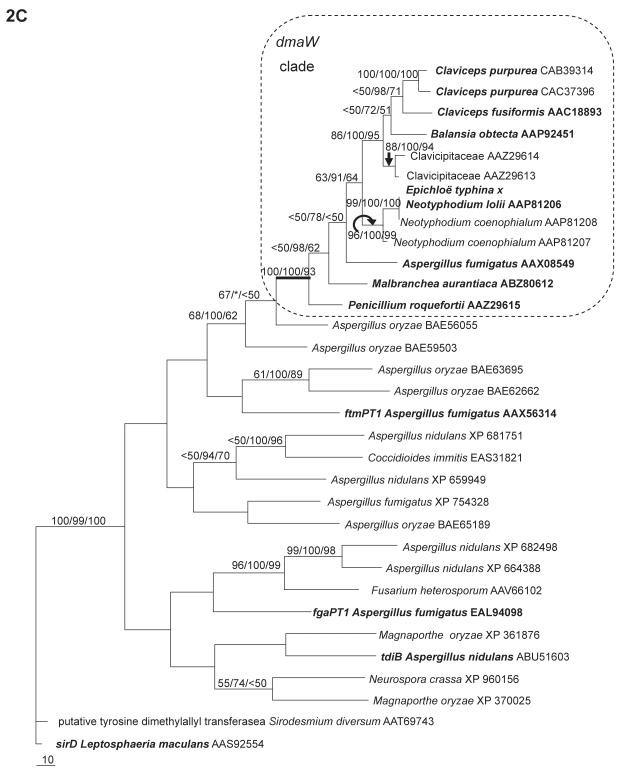
**A**) Strict consensus tree of the six most parsimonious trees for *dmaW* gene products and products of multiple related genes of 34 OTUs based on relatively conserved gene regions. Of the 155 characters, 133 were parsimony-informative; length = 1366, CI = 0.594, RI = 0.499. **B**) One of the two most parsimonious trees of 32 OTUs based on the whole gene region. Of the 933 characters, 456 were parsimony-informative characters. Length = 4974, CI = 0.682, RI = 0.499. **C**) The most parsimonious trees for *dmaW* gene products and products of multiple related genes of 32 OTUs based on relatively conserved gene regions, which were screened by Gblocks. Total aligned characters = 155, informative characters = 135; length = 1427, CI = 0.607, RI = 0.497. OTUs in bold indicate the gene products with functions that have been confirmed. Numbers on branches indicate bootstrap percentage of MP/posterior probabilities of BI/bootstrap percentage of ML; *indicates that particular branch does not exist in the analysis; numbers in circles indicate the possible gene duplication events (also see Fig. 3).

The analysis with conserved gene regions comprised of 34 OTUs resulted in six equally most parsimonious trees, while the whole gene region (933 characters) resulted in eight equally most parsimonious trees. The variations of branch orders among these trees were mainly from the uncertain positions of the two OTUs, the putative DMATrp synthase gene from *Neotyphodium gansuense* and *pax*D *Penicillium paxilli* AAK11526, which were partial sequences. The strict consensus trees of the six trees from conserved regions and eight trees from the whole gene regions showed the same pattern that, in the *dmaW* clade, the two putative DMATrp synthase genes from clavicipitaceous endophyte of convolvulaceous plants (AAZ29613, AAZ29614), the clade comprised of *C. purpurea* and *C. fusiformis dmaW,* as well as the putative DMATrp synthase gene from *N. gansuense* collapsed as a polytomy (Fig. 2A). Outside the *dmaW* clade, *pax*D *P. paxilli* AAK11526 along with the other six OTUs appeared as unresolved branches (Fig. 2A). All trees revealed the same monophyletic group of DMATrp synthase genes (functionally tested) and putative DMATrp synthase genes.

Excluding the two partial sequences (32 OTUs included in analysis) resulted in better resolutions. Both analyses of conserved regions and the whole gene region resulted in two most parsimonious trees. The two trees from whole gene regions differed in the positions of *Magnaporthe oryzae* XP 361876, clade I (*tdiB Aspergillus nidulans* ABU51603, *Neurospora crassa* XP 960156 and *Magnaporthe oryzae* XP 370025) and clade II (*fgaPT1 Aspergillus fumigatus* EAL94098, *Aspergillus oryzae* BAE65189, and *Aspergillus fumigatus* XP 754328) (Fig. 2B). Comparing the phylogenies inferred from the conserved regions and from the entire sequences, within the *dmaW* clade, these trees differed in branching orders of *B. obtecta*, *C. purpurea* and *C. fusiformis dmaW*; and in branch orders of *A. fumigatus* AAX08549, *P. roquefortii* AAZ29615 and *Malbranchea aurantiaca* ABZ80612. All clades with strong statistic support (bootstrapping value >70) were present in all trees (Figs. 2B, C), and all trees clearly indicated monophyly of genes for authentic DMATrp synthase.

Bayesian analyses generally resulted in higher statistical support (posterior probabilities) for the clades having high bootstrap support in parsimony analyses (Figs. 1 and 2) and internal branches. ML analyses resulted tree topologies generally congruent with MP tree, i.e. clades with strong support were congruent with MP (Figs. 1 and 2).

All analyses indicated that conserved gene regions selected by GBlocks did not significantly improve the phylogenetic inference for our data sets.

### Genes associated with *dmaW*-related sequences

Certain *dmaW*-related genes have been identified in gene clusters that are otherwise unrelated to the *EAS* clusters. These include the *sirD* gene of *L. maculans*^23^ and the *paxD* gene of *Penicillium paxilli*.^29^ Because of the close relationship of two *A. oryzae* homologs, and the availability of a complete genome sequence for that fungus,^30^ we checked the genomic contexts of these *A. oryzae* homologs. Neither of them has a comparable gene cluster. In both cases there are a few apparent secondary metabolism genes nearby, but not homologs of the *EAS* cluster genes.

## Discussion

Various profiles of ergot alkaloids (EA) are produced by EA-producing fungi. Evidence for diversification of EA profiles within an individual fungus, as well as among different producers was observed by Panaccione.^7^ The study reported herein is a molecular phylogenetic approach to gain additional insight into the evolution of EA synthesis among fungal producers. Due to its important role in encoding the first step in EA biosynthesis, *dmaW* was used as a marker to infer the evolutionary relationships of the pathways. Our results demonstrate that *dmaW* from *A. fumigatus* and clavicipitaceous fungi formed a monophyletic group indicating that they evolved from a common origin. Therefore we postulate that the *EAS* gene clusters of the two lineages were also from a common origin, which could be a common gene cluster encoding the shared early steps of the pathways of these two lineages. These shared steps might have been present in the most recent common ancestor of the two fungal lineages.

When 34 OTUs were included in the analysis, a hypothetic gene from *P. roqueforti* (GenBank accession number: AAZ29615.^31^ was grouped in the *dmaW* clade. *Penicillium roqueforti* produces the ergot alkaloid isofumigaclavine A,^6^ which would require *dmaW* for its biosynthesis. The product of the *P. roqueforti* gene was 63% identical with the product of *dmaW* of *A. fumigatus*, which was the top match retrieved in a BLAST search with this protein (1e^−112^). These data are consistent with the *P. roqueforti* gene encoding the DMATrp synthase that catalyzes the initial prenylation in ergot alkaloid biosynthesis.

GenBank entries often are annotated completely on the basis of BLAST hits. Since the gene from *C. fusiformis* was identified as encoding DMATrp synthase,^18^ many sequences from other species were annotated as putative dimethylallyltryptophan synthase genes according to the similarity of their sequences to the *C. fusiformis* sequence. However many sequences annotated as dimethylallyltryptophan synthase genes are likely to encode related but nonidentical enzymes catalyzing prenylation or reverse prenylation of different co-substrates or at different positions of the indole rings. Examples of related but functionally different prenyl transferases include the likely tyrosine prenyl transferase in sirodesmin biosynthesis from *L. maculans*,^23^ and the reverse prenyl transferase from *A. fumigatus*.^21^

The rooted tree relating prenyl transferases with known functions showed the most basal separation of DMATrp synthase of *A. fumigatus* with those of the clavicipitaceous fungi. The EA profile of *A. fumigatus* includes a series of clavines, simpler tricyclic or tetracyclic alkaloids. In contrast, clavicipitaceous fungi usually produce more complex EA, ergopeptines and other amides of lysergic acid, in addition to the clavines. It is reasonable that more complex functions were gained along the evolutionary path.

Differences in the arrangement of *EAS* clusters between *A. fumigatus* and clavicipitaceous fungi were likely caused by multiple gene rearrangements through recombinations, deletions and insertions. The *EAS* gene cluster of *A. fumigatus* is in a subtelomeric region.^10^ Frequent recombinations associated with such regions provide a potential explanation for the differences between the *EAS* clusters of *A. fumigatus* and those of the clavicipitaceous fungi.^7^ The chromosomal locations of the clusters in clavicipitaceous species have not yet been determined. Similar rearrangements between more distant genomic locations would account for evolution of clusters, for which selection may favor their inheritance or horizontal transfer as a unit.^32^,^33^ Such rearrangements can be driven by repeats such as retroelements in the fungal genomes, such as observed throughout the ergot alkaloid and lolitrem biosynthesis gene clusters in *Epichloë festucae* and *Neotyphodium lolii*.^34^,^35^

**Figure 3:**
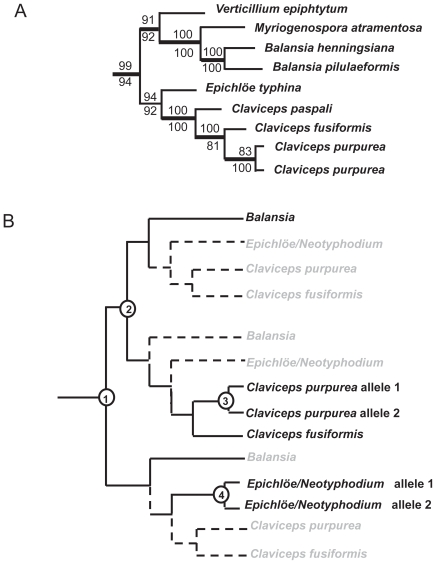
**A**) Partial phylogenetic relationships of species in Clavicipitaceae inferred by MP based on seven gene regions (Tree redrawn from the data of sung et al).^37^ **B**) Diagram indicating the possible gene duplication events that apparently happened in clavicipitaceous *dmaW* lineages. numbers in circles indicate the possible gene duplication events. Dashed lines and shaded OTUs indicate the lost lineages (orthologs).

In fungal systematics, the molecular phylogeny indicates that genus *Claviceps* is more closely related to genus *Epichloë* (asexual stage: *Neotyphodium*) than to *Balansia*;^36^,^37^ (also see Fig. 3A). In our 10 OTU gene trees, the clade of *C. purpurea and C. fusiformis dmaW* was closer to *B. obtecta dmaW* than to *Epichloë* spp. *dmaW* (Fig. 1). The discrepancy between gene tree and species tree can be explained by polymorphic lineage sorting or incomplete sampling.^38^ Once we included more OTUs (34 OTUs and 32 OTUs) in the analyses, the *dmaW* clade separated into two lineages. One lineage was comprised of *C. purpurea* and *C. fusiformis dmaW*, the two putative DMATrp synthase genes from clavicipitaceous endophytes of convolvulaceous plants (AAZ29613, AAZ29614),^31^ the putative DMATrp synthase gene from *N. gansuense*, and the DMATrp synthase gene from *B. obtecta*. The second lineage was comprised of putative DMATrp synthase genes from *N. coenophialum* and *dmaW* from the *E. typhina* × *N. lolii* hybrid. The divergence of these two lineages and the inconsistency of the divergence pattern with species relationships suggest that *dmaW* genes in clavicipitaceous fungi have experienced multiple gene duplications and loss of some copies. An alternative is that there may have been some instances of horizontal gene transfer, but the data are not conclusive in this respect. A scenario involving duplications and losses consistent with the evolutionary relationships of authentic *dmaW* genes is shown in Figure 3B.

## Supplementary Material

Supplement Figure 1 Map of *EAS* clusters from *Claviceps purpurea* strain P1,^14^ *Claviceps fusiformis* strain sD58,^8^ and *Aspergillus fumigatus*.^10^ Arrows indicate the directions of transcription. Black arrows indicate the genes shared in three species; shaded arrows indicate the two genes shared by *C. purpurea* and *C. fusiformis* but not *A. fumigatus*. Lines between maps connect orthologs, and dashed lines indicate the relationships of two *easH* homologues in *C. purpurea* P1 with an apparent pseudogene in *A. fumigatus*.

Supplement Figure 2 Protein sequence alignment screened by GBlocks 0.91b. The selected 155 positions are underlined in blue.

## Figures and Tables

**Table 1:** GenBank numbers and fungal isolates of protein sequences of *dmaW* genes and homologs.

	GenBank number	Fungal species	Isolate	Gene, Function	Reference
1	AAX56314	*Aspergillus fumigatus*	Af293	*ftmPT1* = brevianamide F prenyl transferase	(Grundmann, Li)^21^
2	EAL94098	*Aspergillus fumigatus*	Af293	*fgaPT1* = easL, reverse prenyl transferase	(Unsold, Li)^22^
3	AAX08549	*Aspergillus fumigatus*	Af293	*fgaPT2* = *dmaW*, dimethylallyltryptophan synthase	(Unsold, Li)^40^
4	XP_754328	*Aspergillus fumigatus*	Af293	function unknown	(Nierman et al)^41^
5	ABU51603	*Aspergillus nidulans*	unspecified	tdiB = putative prenyl transferase	(Balibar)^42^
6	XP_681751	*Aspergillus nidulans*	FGSC A4	function unknown	(Galagan et al)^43^
7	XP_682498	*Aspergillus nidulans*	FGSC A4	function unknown	(Galagan et al)^43^
8	XP_659949	*Aspergillus nidulans*	FGSC A4	function unknown	(Galagan et al)^43^
9	XP_664388	*Aspergillus nidulans*	FGSC A4	function unknown	(Galagan et al)^43^
10	BAE56055	*Aspergillus oryzae*	RIB 40	function unknown	(Machida et al)^31^
11	BAE59503	*Aspergillus oryzae*	RIB 40	function unknown	(Machida et al)^31^
12	BAE62662	*Aspergillus oryzae*	RIB 40	function unknown	(Machida et al)^31^
13	BAE65189	*Aspergillus oryzae*	RIB 4	function unknown	(Machida et al)^31^
14	BAE63695	*Aspergillus oryzae*	RIB 4	function unknown	(Machida et al)^31^
15	AAP92451	*Balansia obtecta*	B249	*dmaW* = dimethylallyltryptophan synthase	(Wang et al)^15^
16	AAC18893	*Claviceps fusiformis*	ATCC26245	*dmaW* = dimethylallyltryptophan synthase	(Tsai et al)^19^
17	CAB39314	*Claviceps purpurea*	P1	*dmaW* = *cpd1*, dimethylallyltryptophan synthase allele 1	(Tudzynski et al)^14^
18	CAC37396	*Claviceps purpurea*	T5	*dmaW* = *cpd2*, putative dimethylallyltryptophan synthase allele 2	(Arntz and Tudzynski, unpublished)
19	AAZ29613	*Clavicipitaceae*	US2005a	*dmaW* = putative dimethylallyltryptophan synthase	(Steiner et al)^32^
20	AAZ29614	*Clavicipitaceae*	US2005b	*dmaW* = putative dimethylallyltryptophan synthase	(Markert et al)^43^
21	EAS31821	*Coccidioides immitis*	RS	function unknown	(Birren et al, unpublished)
22	AAP81206	*Epichloë typhina* × *Neotyphodium lolii*	Lp1	*dmaW* = dimethylallyltryptophan synthase	(Wang et al)^15^
23	AAV66102	*Fusarium heterosporum*	ATCC74349	function unknown	(Sims et al)^44^
24	AAS92554	*Leptosphaeria maculans*	ICBN 18	*sirD* = putative tyrosine dimethylallyl transferase	(Gardiner et al)^24^
25	XP_361876	*Magnaporthe grisea*	70-15	function unknown	(Dean et al)^45^
26	XP_370025	*Magnaporthe grisea*	70-15	function unknown	(Dean et al)^46^
27	ABZ80612	*Malbranchea aurantiaca*	RRC1813	*MaPT* = dmaW, dimethylallyltryptophan synthase	(Ding et al)^47^
28	AAP81207	*Neotyphodium coenophialum*	ATCC90664	*dmaW* = dimethylallyltryptophan synthase allele 1	(Wang et al)^15^
29	AAP81208	*Neotyphodium coenophialum*	ATCC90664	*dmaW* = dimethylallyltryptophan synthase allele 2	(Wang et al)^15^
30	none	*Neotyphodium gansuense*	E818	*dmaW* = putative dimethylallyltryptophan synthase	Unpublished data
31	XP_960156	*Neurospora crassa*	OR74A	function unknown	(Galagan et al)^48^
32	AAZ29615	*Penicillium roqueforti*	IasaF09	*dmaW* = putative dimethylallyltryptophan synthase	(Steiner et al)^49^
33	AAK11526	*Penicillium paxilli*	ATCC26601	*paxD* = function unknown	(Young et al)^30^
34	AAT69743	*Sirodesmium diversum*	ATCC36539	putative dimethylallyltyrosine synthase	Gardiner and Howlett, unpublished

**Table 2:** Fitness of the trees resulting from maximum parsimony searches with different matrix scoring settings.

Strategy	Scoring matrix	Number of trees	Length	CI	RI	RC	HI	G-fit
	BLOSUM30	1	5252	0.653	0.491	0.32	0.347	−224.198
	BLOSUM45	4	5214	0.64	0.486	0.311	0.36	−219.496
FFT-NS-i	BLOSUM62	6	5230	0.637	0.491	0.313	0.363	−208.403
	BLOSUM80	8	5170	0.638	0.487	0.31	0.362	−207.579
	JTT100	5	5187	0.646	0.494	0.319	0.354	−212.253
	JTT200	2	5193	0.662	0.491	0.325	0.338	−235.767
	BLOSUM30	1	5200	0.636	0.486	0.309	0.364	−211.530
	BLOSUM45	6	5185	0.636	0.491	0.312	0.364	−203.114
E-INS-i	BLOSUM62	6	5190	0.637	0.489	0.312	0.363	−205.306
	BLOSUM80	1	5203	0.635	0.488	0.310	0.365	−204.704
	JTT100	2	5229	0.631	0.483	0.305	0.369	−206.307
	JTT200	3	5174	0.632	0.486	0.307	0.368	−204.534

**Table 3:** Fitness of the trees resulting from maximum parsimony searches with different gap opening penalties (OP) and gap extension penalties (OF) (FFT-NS-i, JTT200).

OP	OF	Number of trees	Length	CI	RI	RC	HI	G-fit
1.0	0	3	5098	0.675	0.498	0.336	0.325	−245.815
1.0	0.5	1	5219	0.628	0.483	0.303	0.372	−206.925
1.0	1	1	5217	0.625	0.479	0.3	0.375	−197.856
2.0	0	11	5227	0.649	0.489	0.317	0.351	−226.988
2.0	0.5	4	5281	0.632	0.485	0.306	0.368	−206.599
2.0	1	1	5272	0.637	0.489	0.312	0.363	−204.882
3.0	0	7	5354	0.658	0.491	0.323	0.342	−239.881
3.0	0.5	4	5280	0.64	0.49	0.314	0.36	−210.476
3.0	1	12	5317	0.639	0.483	0.308	0.361	−206.114

## References

[1] Keller U Tudzynski P Osiewacz HD 2002 Ergot Alkaloids The Mycota: Industrial Applications Springer Berlin

[2] Schardl CL Panaccione DG Tudzynski P Ergot alkaloids—biology and molecular biology The Alkaloids Chemistry and Biology 2006 45 86 Academic Press 10.1016/s1099-4831(06)63002-2 17133714

[3] Tudzynski P Correia T Keller U Biotechnology and genetics of ergot alkaloids Applied Microbiology and Biotechnology 2001 57 593 605 11778866 10.1007/s002530100801

[4] Panaccione DG Coyle CM Abundant respirable ergot alkaloids from the common airborne fungus *Aspergillus fumigatus* Applied and Environmental Microbiology 2005 71 3106 11 15933008 10.1128/AEM.71.6.3106-3111.2005 PMC1151833

[5] Kozlovsky AG Kren V Cvak L Producers of ergot alkaloids out of *Claviceps* genus Ergot: The Genus *Claviceps* 1999 479 99 Harwood Academic Publishers The Netherlands

[6] Scott PM Merrian M-A Polonsky J Roquefortin and isofumigaclavine A, metabolites from *Penicillium roqueforti* Experientia 1976 32 140 2

[7] Panaccione DG Origins and significance of ergot alkaloid diversity in fungi FEMS Microbiology Letters 2005 251 9 17 16112823 10.1016/j.femsle.2005.07.039

[8] Lorenz N Wilson EV Machado C Schardl CL Tudzynski P Comparison of ergot alkaloid biosynthesis gene clusters in *Claviceps* species indicates loss of late pathway steps in evolution of *C. fusiformis* Applied Environmental Microbiology 2007 73 7185 91 10.1128/AEM.01040-07 PMC2168186 17720822

[9] Arntz C Tudzynski P Identification of genes induced in alkaloid-producing cultures of *Claviceps* sp Current Genetics 1997 31 357 60 9108144 10.1007/s002940050216

[10] Coyle CM Panaccione DG An ergot alkaloid biosynthesis gene and clustered hypothetical genes from *Aspergillus fumigatus* Applied and Environmental Microbiology 2005 71 3112 8 15933009 10.1128/AEM.71.6.3112-3118.2005 PMC1151871

[11] Damrongkool P Sedlock AB Young CA Structural analysis of a peptide synthetase gene required for ergotpeptine production in the endophytic fungus*Neotyphodium lolii* DNA Sequence 2005 16 379 85 16243728 10.1080/10425170500273005

[12] Haarmann T Machado C Lubbe Y The ergot alkaloid gene cluster in *Claviceps purpurea*: Extension of the cluster sequence and intra species evolution Phytochemistry 2005 66 1312 20 15904941 10.1016/j.phytochem.2005.04.011

[13] Panaccione DG Johnson RD Wang J Elimination of ergovaline from a grass-*Neotyphodium* endophyte symbiosis by genetic modification of the endophyte Proceedings of the National Academy of Sciences of the United States of America 2001 98 12820 5 11592979 10.1073/pnas.221198698 PMC60137

[14] Tudzynski P Holter K Correia T Evidence for an ergot alkaloid gene cluster in *Claviceps purpurea* Molecular and General Genetics 1999 261 133 141 10071219 10.1007/s004380050950

[15] Wang J Machado C Panaccione DG Tsai HF Schardl CL The determinant step in ergot alkaloid biosynthesis by an endophyte of perennial ryegrass Fungal Genetics and Biology 2004 41 189 98 14732265 10.1016/j.fgb.2003.10.002

[16] Vazquez MJ Roa AM Reyes F A novel ergot alkaloid as a 5-HT(1A) inhibitor produced by *Dicyma* sp Journal of Medicinal Chemistry 2003 46 5117 20 14613313 10.1021/jm0341204

[17] Floss HG Robbers JE Heinstein PF Regulatory control mechanisms in alkaloid biosynthesis Recent Advances in Phytochemistry 1974 8 141 78

[18] Krupinski VM Robbers JE Floss HG Physiological study of ergot: induction of alkaloid synthesis by tryptophan at enzymatic level Journal of Bacteriology 1976 125 158 65 1372 10.1128/jb.125.1.158-165.1976 PMC233347

[19] Tsai HF Wang H Gebler JC Poulter CD Schardl CL The *Claviceps purpurea* gene encoding dimethylallytryptophan synthase, the committed step for ergot alkaloid biosynthesis Biochemical and Biophysical Research Communications 1995 216 119 25 7488077 10.1006/bbrc.1995.2599

[20] Machado C Studies of ergot alkaloid biosynthesis genes in clavicipitaceous fungi University of Kentucky 2004

[21] Grundmann A Li SM Overproduction, purification and characterization of FtmPT1, a brevianamide F prenyltransferase from *Aspergillus fumigatus* Microbiology 2005 151 2199 07 16000710 10.1099/mic.0.27962-0

[22] Unsold IA Li SM Reverse prenyltransferase in the biosynthesis of fumigaclavine C in *Aspergillus fumigatus*: gene expression, purification, and characterization of fumigaclavine C synthase FGAPT1 Chembiochem 2006 158 64 16397874 10.1002/cbic.200500318

[23] Bok JW Hoffmeister D Maggio-Hall LA Genomic mining for *Aspergillus* natural products Chemistry and Biology 2006 13 31 7 16426969 10.1016/j.chembiol.2005.10.008

[24] Gardiner DM Cozijnsen AJ Wilson LM Pedras MSC Howlett BJ The sirodesmin biosynthetic gene cluster of the plant pathogenic fungus *Leptosphaeria maculans* Molecular Microbiology 2004 53 1307 18 15387811 10.1111/j.1365-2958.2004.04215.x

[25] Katoh K Kuma Ki Toh H Miyata T MAFFT version 5: improvement in accuracy of multiple sequence alignment Nucleic Acids Research 2005 33 511 8 15661851 10.1093/nar/gki198 PMC548345

[26] Castresana J Selection of conserved blocks from multiple alignments for their use in phylogenetic analysis Molecular Biology and Evolution 2000 17 540 2 10742046 10.1093/oxfordjournals.molbev.a026334

[27] Swofford DL PAUP*. Phylogenetic Analysis Using Parsimony (*and other methods). Version 4 (computer program) Sinauer Associates, Sunderland, Massachusetts 1998

[28] Huelsenbeck JP Ronquist F MRBAYES: Bayesian inference of phylogeny Bioinformatics 2001 17 754 5 11524383 10.1093/bioinformatics/17.8.754

[29] Guindon S Lethiec F Duroux P Gascuel O PHYML online—a web server for fast maximum likelihood-based phylogenetic inference Nucleic Acids Research 2005 33 web server issue W557 9 15980534 10.1093/nar/gki352 PMC1160113

[30] Young C McMillan L Telfer E Scott B Molecular cloning and genetic analysis of an indole-diterpene gene cluster from *Penicillium paxilli* Molecular Microbiology 2001 39 754 64 11169115 10.1046/j.1365-2958.2001.02265.x

[31] Machida M Asai K Sano M Genome sequencing and analysis of *Aspergillus oryzae* Nature 2005 438 1157 61 16372010 10.1038/nature04300

[32] Steiner U Ahimsa-Müller MA Markert A Molecular characterization of a seed transmitted clavicipitaceous fungus occurring on dicotyledoneous plants (Convolvulaceae) Planta 2006 224 533 44 16525783 10.1007/s00425-006-0241-0

[33] Carbone I Ramirez-Prado J Jakobek J Horn B Gene duplication, modularity and adaptation in the evolution of the aflatoxin gene cluster BMC Evolutionary Biology 2007 7 111 17620135 10.1186/1471-2148-7-111 PMC1949824

[34] Walton J Horizontal gene transfer and the evolution of secondary metabolite gene clusters in fungi: an hypothesis Fungal Genetics and Biology 2000 30 167 71 11035938 10.1006/fgbi.2000.1224

[35] Fleetwood D Scott B Lane G Tanaka A Johnson R A complex ergovaline gene cluster in *Epichloë* endophytes of grasses Applied and Environmental Microbiology 2007 73 2571 9 17308187 10.1128/AEM.00257-07 PMC1855613

[36] Young C Tapper B May K Moon C Schardl C Scott B Indole-diterpene biosynthetic capability of *Epichloë* endophytes as predicted by *ltm* gene analysis Applied and Environmental Microbiology 2009 75 2200 11 19181837 10.1128/AEM.00953-08 PMC2663189

[37] Sung GH Sung JM Hywel-Jones NL Spatafora JW A multi-gene phylogeny of Clavicipitaceae (Ascomycota, Fungi): Identification of localized incongruence using a combinational bootstrap approach Molecular Phylogenetics and Evolution 2007 44 1204 23 17555990 10.1016/j.ympev.2007.03.011

[38] Lewis EA Sullivan RF White JF Jr James F White J Bacon CW Hywel-Jones NL Spatafora JW Distinguishing features of the tribe Balansieae Clavicipitalean Fungi: evolutionary biology, chemistry, biocontrol, and cultural impacts 2003 151 67 Marcel Dekker, Inc New York, Basel

[39] Doyle JJ Gene trees and species trees: molecular systematics as one-character taxonomy Systematic Botany 1992 17 144 63

[40] Unsold IA Li SM Overproduction, purification and characterization of FgaPT2, a dimethylallyltryptophan synthase from *Aspergillus fumigatus* Microbiology 2005 151 1499 505 15870460 10.1099/mic.0.27759-0

[41] Nierman WC Pain A Anderson MJ Genomic sequence of the pathogenic and allergenic filamentous fungus *Aspergillus fumigatus* Nature 2005 438 1151 6 16372009 10.1038/nature04332

[42] Balibar CJ Howard-Jone AR Walsh CT Terrequinone A biosynthesis through L-tryptophan oxidation, dimerization and bisprenylation Nature Chemical Biology 2007 3 584 92 10.1038/nchembio.2007.20 17704773

[43] Galagan JE Calvo SE Cuomo C Sequencing of *Aspergillus nidulans* and comparative analysis with *A. fumigatus* and *A. oryzae* Nature 2005 438 1105 5 16372000 10.1038/nature04341

[44] Markert A Steffan N Ploss K Biosynthesis and accumulation of ergoline alkaloids in a mutualistic association between *Ipomoea asarifolia* (Convolvulaceae) and a *clavicipitalean fungus* Plant Physiology 2008 147 296 05 18344419 10.1104/pp.108.116699 PMC2330284

[45] Sims JW Fillmore JP Warner DD Schmidt EW Equisetin biosynthesis in *Fusarium heterosporum* Chemical Communications 2005 186 7 15724180 10.1039/b413523g

[46] Dean RA Talbot NJ Ebbole DJ The genome sequence of the rice blast fungus *Magnaporthe grisea* Nature 2005 434 980 6 15846337 10.1038/nature03449

[47] Ding Y Williams RM Sherman DH Molecular analysis of a 4-dimethylallyltryptophan synthase from *Malbranchea aurantiaca* Journal of Biological Chemistry 2008 283 16068 76 18390548 10.1074/jbc.M801991200 PMC2414270

[48] Galagan JE Calvo SE Borkovich KA The genome sequence of the filamentous fungus *Neurospora crassa* Nature 2003 422 859 68 12712197 10.1038/nature01554

[49] Steiner U Hellwig S Leistner E Specificity in the interaction between an epibiotic clavicipitalean fungus and its convolvulaceous host in a fungus/plant symbiotum Plant Signaling and Behavior 2008 3 704 6 19704834 10.4161/psb.3.9.6432 PMC2634565

