# Supplementary figures and images for: Phylogenetic Analyses Reveal Monophyletic Origin of the Ergot Alkaloid Gene dmaW in Fungi
Source: Evol Bioinform Online. 2009 Jun 4;5:15–30. doi: 10.4137/ebo.s2633 (PMC2747131; doi:10.4137/ebo.s2633)

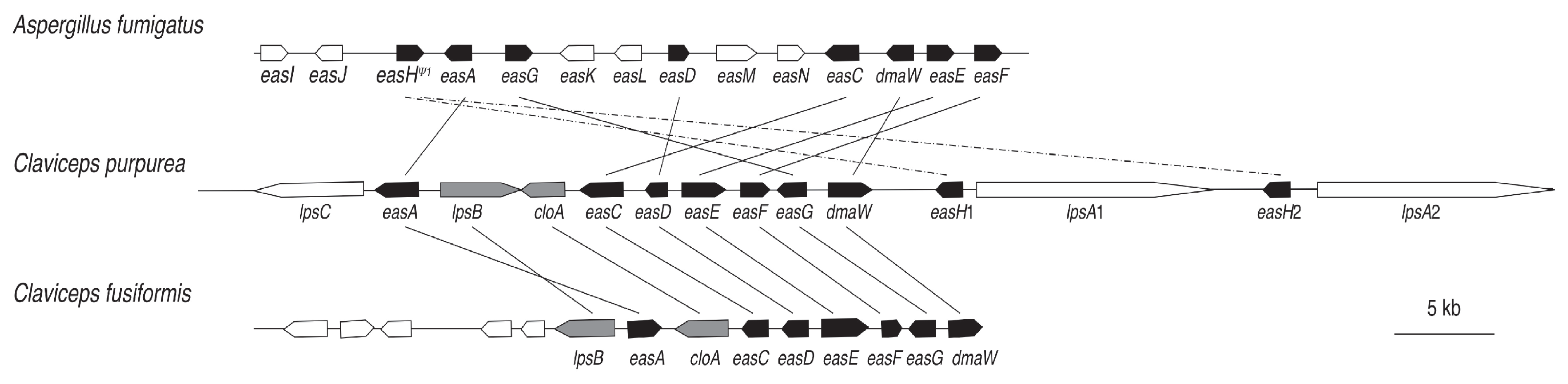

Supplement: Supplement Figure 1 — Map of EAS clusters from Claviceps purpurea strain P1,14 Claviceps fusiformis strain sD58,8 and Aspergillus fumigatus.10 Arrows indicate the directions of transcription. Black arrows indicate the genes shared in three species; shaded arrows indicate the two genes shared by C. purpurea and C. fusiformis but not A. fumigatus. Lines between maps connect orthologs, and dashed lines indicate the relationships of two easH homologues in C. purpurea P1 with an apparent pseudogene in A. fumigatus. [file ebo-2009-015f4.tif]

## Slide 1
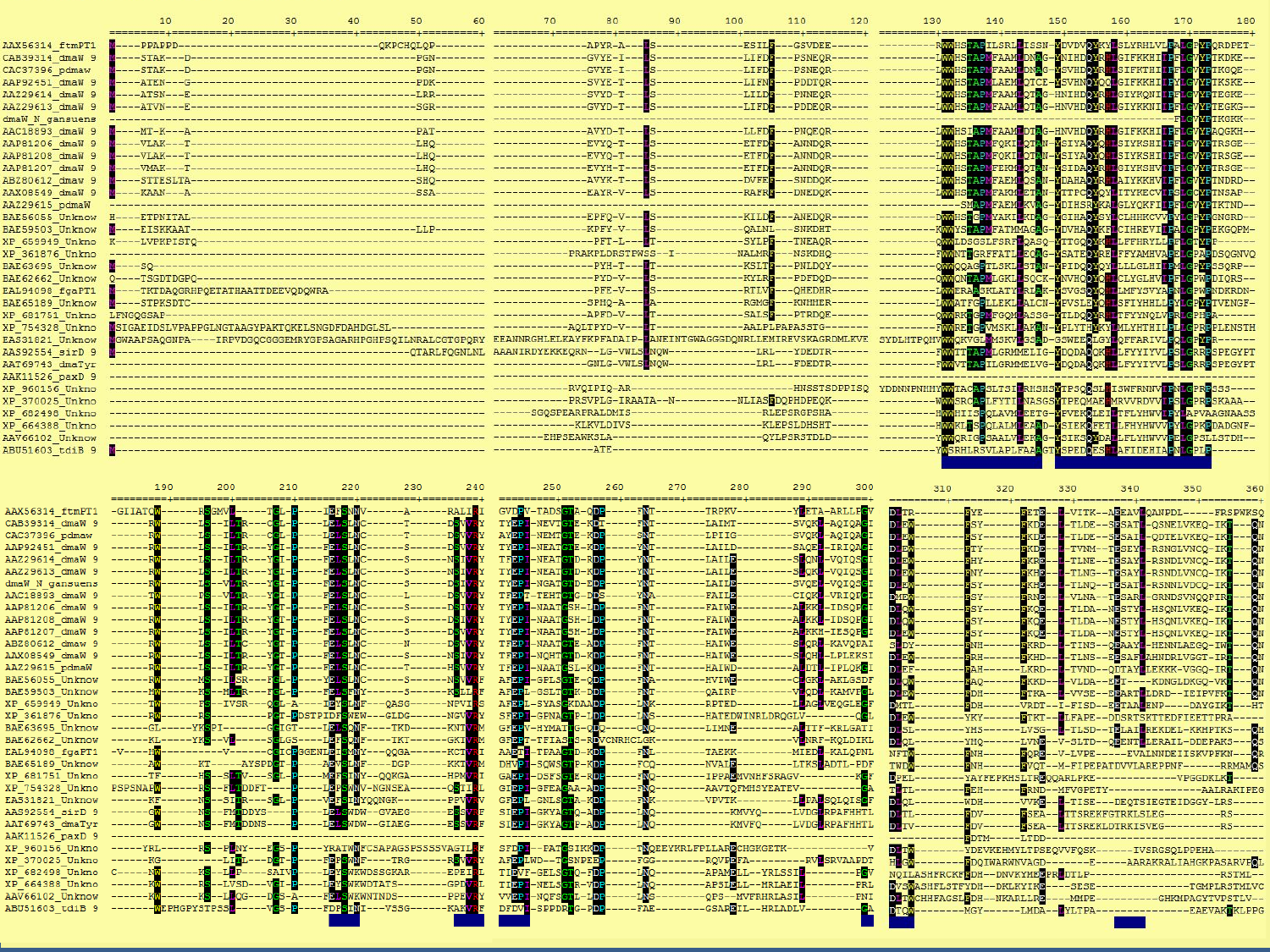

## Slide 2
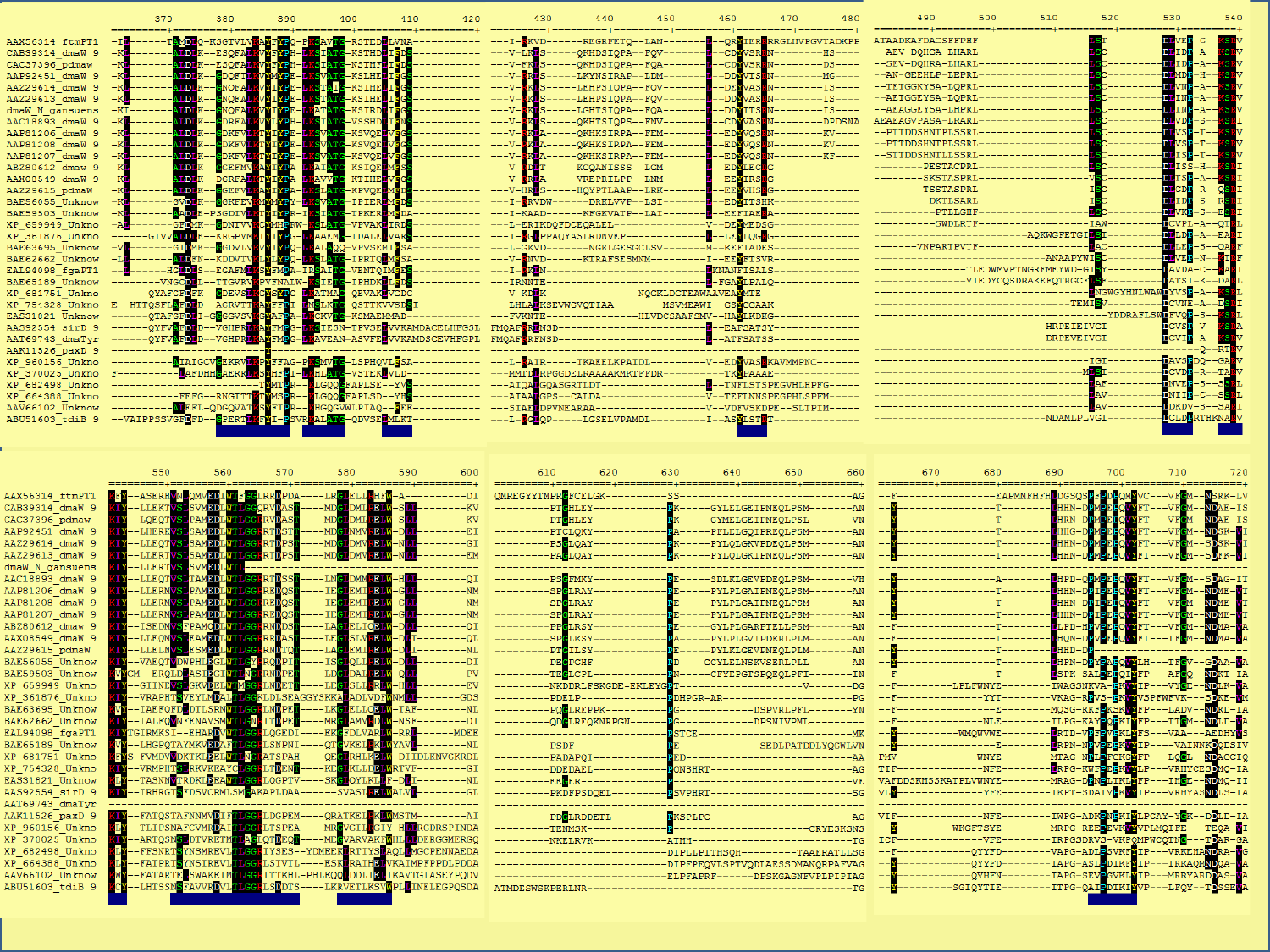

## Slide 3
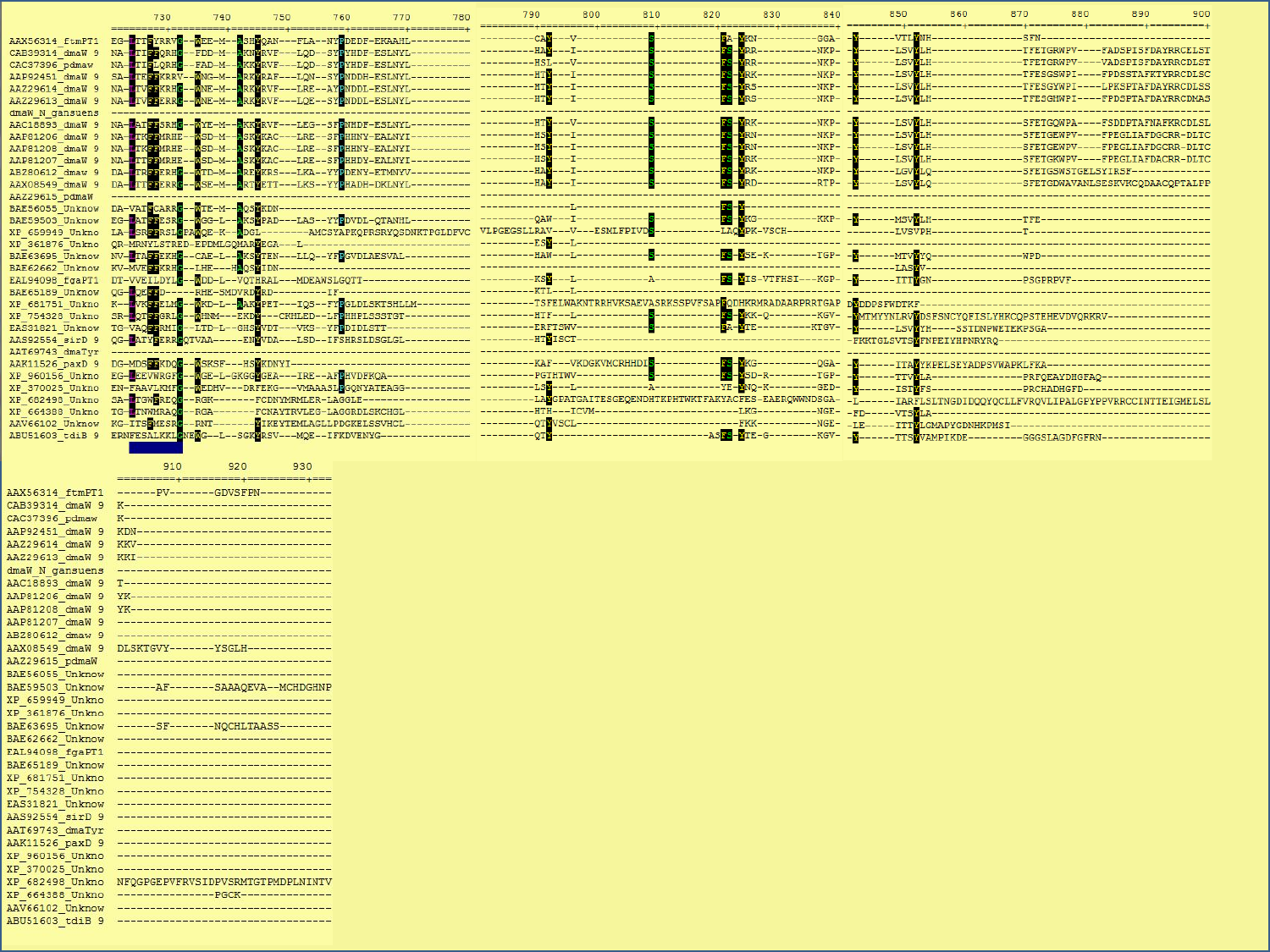

Supplement: Supplement Figure 2 — Protein sequence alignment screened by GBlocks 0.91b. The selected 155 positions are underlined in blue. [file Supplement_Figure_2_in_PDF.ppt]
